# Gallbladder osteoma in a 66-year-old female; Case report and review of literature^☆^

**DOI:** 10.1016/j.ijscr.2020.03.038

**Published:** 2020-04-01

**Authors:** Alberto Valdes Castañeda, Raul Alexander Cuevas Bustos, Moises Brener Chaoul, Marcos Jafif Cojab, Juan Pablo Arribas Martin, Carlos Mancera Steiner, Diego L. Jorge, Maria Veronica Velasco Vales, Oscar Cervantes Gutierrez, Angel Flores-Huidobro Martinez, Felix Alejandro Perez Tristan

**Affiliations:** a Hospital Angeles Lomas, Department of Surgery, Edo. Mex 52763, Mexico; b Hospital Angeles Lomas, Department of Pathology, Edo. Mex 52763, Mexico

**Keywords:** Osteoma, Gallbladder, Rare case, Mesenchymal tumors, Incidental lesions

## Abstract

**Introduction:**

Gallbladder mesenchymal tumours are rare. The more common include fibroma, lipoma or haemangioma. A gallbladder osteoma is very rare indeed there is only one other case reported in medical literature). We report a new case.

**Presentation of case:**

A 66-year-old female presented to the emergency department complaining of colicky epigastric pain and generalised abdominal discomfort for 1 month. The pain was scored 5/10 but there were no associated symptoms of fever, nausea or vomiting. Vital signs were normal as were all laboratory parameters. An abdominal ultrasound revealed a thin walled gallbladder with a solitary 3 mm polyp. Motility studies confirmed gallbladder dyskinesia.

Laboratory studies were ordered reporting normal findings. Abdominal ultrasound was ordered reporting an image suggesting a gallbladder polyp and gallbladder dyskinesia. Cholecystectomy was done without any incidents and the gallbladder was sent to pathology. Pathology reported mature bone tissue in the stromal gallbladder tissue. Due to the rareness of the presence of mature bone tissue in the gallbladder we decided to report the case for the medical community.

**Discussion:**

Osteomas are mesenchymal cell tumors derived from the mesoderm; the gallbladder may be the primary site of numerous types of mesenchymal tumors, although these tumors are common the location is not.

**Conclusions:**

We report only the second case of gallbladder osteoma. These mesenchymal tumours are common but this location is not. This case report will serve to remind readers of both benign osteomas and unusual conditions causing gallbladder disease.

## Highlights

• Gallbladder osteomas are mesenchymal derived tumors.
• Account for an extraordinary low incidental finding during routine cholecystectomy.
• There is only one other case reported in medical literature.

## 1 Introduction

Gallbladder mesenchymal tumours are rare. The more common include fibroma, lipoma or haemangioma. A gallbladder osteoma is very rare indeed there is only one other case reported in medical literature). We report a new case.

## 2 Presentation of case

**Fig. 1:**
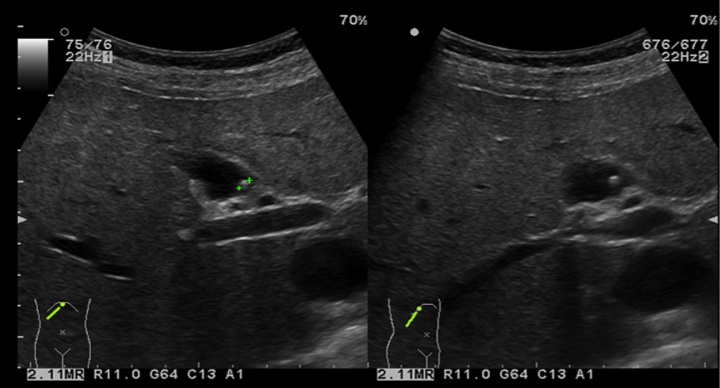
Abdominal USG showing a polypoid lesion inside the gallbladder; later on the lesion was confirmed to be an osteoma.

**Fig. 2:**
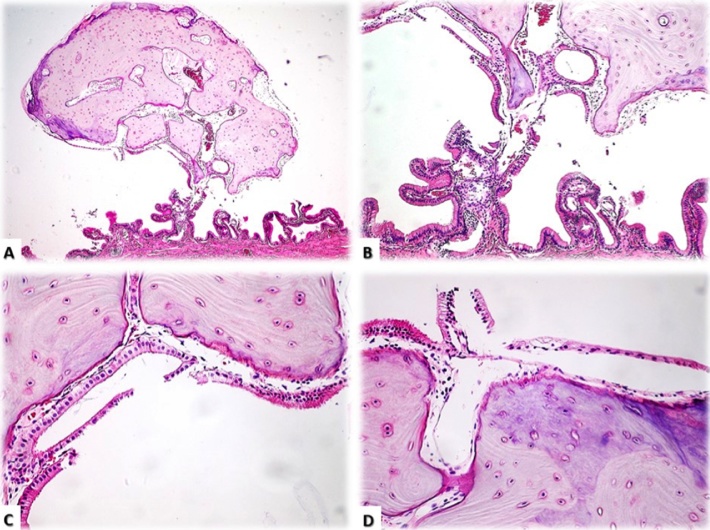
Histopathological study (H & E stain): A.- Panoramic view (4×) of polypoid lesion dependent on the mucosa of the gall bladder, at neck level. B.- Pedicle of the lesion in continuity with the lamina propria of the mucosa (10×). C and D.- Central portion of the lesion shows mature bone tissue of cortical appearance, with simple cylindrical, vesicular, discontinuous epithelial lining on the periphery of the polyp (40×).

A 66-year-old female presented to the emergency department complaining of colicky epigastric pain and generalised abdominal discomfort for 1 month. The pain was scored 5/10 but there were no associated symptoms of fever, nausea or vomiting. Vital signs were normal as were all laboratory parameters. An abdominal ultrasound revealed a thin walled gallbladder with a solitary 3 mm polyp (Fig. 1). Motility studies confirmed gallbladder dyskinesia. A laparoscopic cholecystectomy was undertaken and the gallbladder sent for histology and pathology for further studies. Histopathology reported a healthy, non-inflamed gallbladder and a solitary 3 mm polypoid lesion pedicled towards the neck. Features of this polyp were of mature lamellar type bone tissue, without the presence of bone marrow cells or osteoblasts in the periphery, covered by biliary type epithelium (Fig. 2). The remaining gallbladder was normal and there was no evidence of cholesterolosis or gallstones. The final report concluded this was a benign gallbladder osteoma.

## 3 Discussion

Osteomas are mesenchymal cell tumours derived from the mesoderm; the gallbladder may be the primary site of numerous types of mesenchymal tumours, although these tumours are rare. Benign tumours include lipomas, neurofibromas, lymphangiomas, hemangiomas, among others. Most are small, solitary lesions that are often asymptomatic and detected as incidental lesions in the setting of cholecystectomy for other reasons. Osteoma is a benign bone-forming tumor with hallmark of tumor cells directly forming mature bone. O Osteoma accounts for around 5% of all bone tumors and 11% of benign bone tumors with a male predilection. It occurs predominantly in long bones of the appendicular skeleton.

Osteomas are very rarely found in the gallbladder and there is only one previous case reported in the literature [1]. An extensive research to find similar cases around the medical literature showed us how rare this entity is, finding only one case report of a gallbladder osteoma [[1], [2], [3], [4], [5]].

## 4 Conclusions

We report only the second case of gallbladder osteoma. These mesenchymal tumours are common but this location is not. This case report will serve to remind readers of both benign osteomas and unusual conditions causing gallbladder disease.

## Conflicts of interest

There was no conflict of interest.

## Sources of funding

There was no sponsorship for this study.

## Ethical approval

There was no need for ethical approval.

## Consent

Written informed consent was obtained from the patient for publication of this case report and accompanying images. A copy of the written consent is available for review by the Editor-in-Chief of this journal on request.

## Author contribution

Alberto; Valdes Castañeda MD FACS: Study design.

Juan Pablo; Arribas Martin MD: Study design.

Carlos; Mancera Steiner MD: Study design.

Marcos; Jafif Cojab MD: Data collection, data analysis, writing the paper.

Raul Alexander; Cuevas Bustos MD: Data collection, data analysis.

Jorge, Diego MD: Data collection, data analysis.

Moises Brener Chaoul MD: Data collection, data analysis.

Maria Veronica Velasco Vales MD: Data collection, data analysis.

Oscar Cervantes Gutierrez MD: Data collection, data analysis.

Felix Alejandro Perez Trsitan MD: Data collection, data analysis.

Angel Flores-Huidobro Martinez MD: Data collection, data analysis.

## Registration of research studies

NA.

## Guarantor

Alberto; Valdes Castañeda MD FACS.

## Provenance and peer review

Not commissioned, externally peer-reviewed.
